# Pannexin channels in inflammation and tumorigenesis

**DOI:** 10.3389/fcell.2025.1647765

**Published:** 2025-08-20

**Authors:** Mengmeng Jiang, Xiaojia Li, Keping Xie

**Affiliations:** ^1^ Center for Pancreatic Cancer Research, The South China University of Technology School of Medicine, Guangzhou, China; ^2^ The South China University of Technology Comprehensive Cancer Center, Guangzhou, Guangdong, China

**Keywords:** pannexin, fibrosis, inflammation, tumorigenesis, therapeutics

## Abstract

Pannexin (Panx) channels are oligomeric heptamers of PANX proteins, comprising Panx1, Panx2 and Panx3. These channels facilitate the extracellular release of signaling molecules up to 1.5 kDa in size, including adenosine triphosphate (ATP), amino acids, ions, and other metabolites. These signaling molecules can activate receptors either on their cells of origin or neighboring cells, triggering downstream signaling cascades that mediate various physiological responses. Current pharmacological inhibitors of Panx channels include Food and Drug Administration (FDA)-approved drugs such as Carbenoxolone (CBX), Probenecid (PBN), and Spironolactone, along with chemically synthetic compounds 10Panx. Both genetic modulation of Panx expression and pharmacological manipulation have demonstrated the channels’ critical involvement in various human pathologies, establishing them as promising therapeutic targets for clinical intervention. In this review, we will specifically examine the signaling regulatory functions of Panx channels in the processes of inflammation and tumorigenesis; systematically evaluate the therapeutic potential of Panx inhibitors in these pathological contexts, critically analyze current research limitations, and strategically propose future perspectives in Panx channels and its inhibitors research.

## Introduction

Gap junctions are intercellular channels that directly connect the cytoplasm of adjacent cells in multicellular organisms, a conserved feature across both vertebrate and invertebrate species (Larsen, 1977). These specialized channels permit the bidirectional exchange of ions, second messengers (<1.5 kDa), and metabolic substrates, thereby coordinating multicellular activities (Hertzberg et al., 1981). In vertebrates, gap junction channels are composed of Connexin (Cx) proteins that assemble into hexameric complexes (Connexons) spanning adjacent plasma membranes (Bruzzone et al., 1996). Invertebrates utilize structurally analogous Innexin proteins to form functional gap junctions, despite lacking sequence homology with connexins (Phelan et al., 1998). The subsequent discovery of Pannexins (Panx), vertebrate homologs of invertebrate Innexins, reveals a distinct family of large-pore channels (Panchin et al., 2000). The nomenclature reflects both their widespread tissue distribution (from Greek “pan” meaning “all”) and channel-forming capacity (from Latin “nexus” denoting “connection”) (Panchin et al., 2000).

Unlike connexons that require docking between two hemi-channels for intercellular communication (Bruzzone et al., 1996; Laird, 1996), Panx channels typically function as single-membrane hemi-channels that mediate autocrine/paracrine signaling through ATP release and other small molecule efflux (Bao et al., 2004). However, recent evidence indicates that Panx1 can also form functional intercellular cell-cell channels, exhibiting characteristic voltage-dependent properties (Palacios-Prado et al., 2022). In this review, while acknowledging this dual functionality, our primary focus remains on its hemichannel roles across the discussed physiological and pathological contexts.

Accumulating evidence positions Panx channels as key regulators of organogenesis, tissue repair, hormonal regulation, and programmed cell death. Consequently, Panx dysregulation has been mechanistically linked to multiple disease states, with particularly strong associations emerging in inflammatory pathologies and tumorigenesis.

This review will systematically examine the structure, localization, physiological and pathophysiological functions of Panx channels. We will particularly emphasize the recent advances in pharmacological targeting of Panx channels using clinically approved drugs (e.g., CBX, PBN) and synthetic peptide inhibitor targeting Panx1 W74 to Y83 (10Panx), and evaluate their therapeutic potential in inflammation and cancer.

## Panx proteins structure, localization and physiological function

**FIGURE 1:**
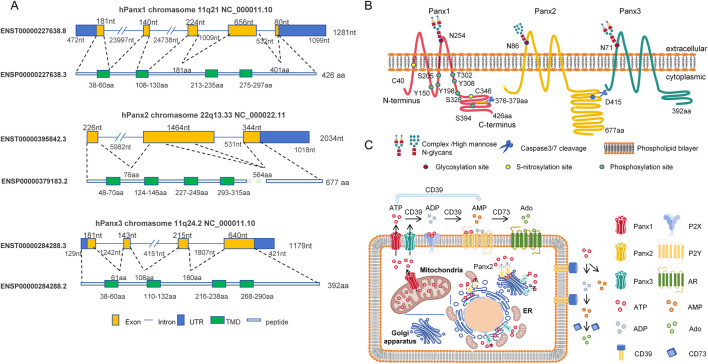
Schematic diagram of structural and functional features of the human pannexin channel protein family. **(A)** Genomic organization: Chromosomal localization and CCDS-annotated structures of Panx1 (11q21), Panx2 (22q13.33), and Panx3 (11q24.2) genes. Exons (yellow rectangles) and introns (blue lines) are shown to scale. Green segments denote exons encoding transmembrane domains (TMD). Untranslated regions (UTR) are indicated by blue rectangles. **(B)** Protein topology: Four-pass transmembrane topology of Panx proteins in phospholipid bilayer. Key post-translational modification sites are annotated: Glycosylation (red circles), Phosphorylation (orange circles), S-nitrosylation (yellow circles), and Caspase cleavage (blue scissors). **(C)** Subcellular localization and function: ATP release pathways mediated by Panx1 (plasma membrane/mitochondria), Panx2 (endoplasmic reticulum [ER], Golgi apparatus, and ER-Golgi membrane contact sites), and Panx3 (plasma membrane/ER/Golgi). Extracellular ATP hydrolysis by CD39 (ATP/ADP→AMP) and CD73 (AMP→adenosine) activates purinergic signaling cascades.

Above all, to provide an integrated overview of Panx channels, we constructed a schematic (Figure 1) detailing their genomic organization, structural topology, subcellular localization, and physiological functions.

Diagram depicting the structure and functions of Panx1, Panx2, and Panx3 proteins. Panel A: Displays exon, intron, UTR, TMD, and peptide structures with nucleotide/amino acid lengths on chromosomes 11q21 (Panx1), 22q13 (Panx2), and 11q24 (Panx3). Panel B: Illustrates protein localization within the cell membrane, showing post-translational modifications and cleavage sites. Panel C: Shows subcellular distributions of Panx1 ((plasmamembrane/ mitochondria), Panx2 (ER, Golgi and ER-Golgi membrane contact sites), and Panx3 (plasma membrane/ER/Golgi). Highlights extracellular ATP hydrolysis by CD39/CD73 enzymes into ADP, AMP, and Ado, which interact with P2X, P2Y, and Ado receptors.

### Structure

The mammalian pannexin family comprises three distinct homologs: Panx1, Panx2, and Panx3. Genetic mapping reveals that both Panx1 and Panx3 reside on human chromosome 11, encoding proteins with molecular masses of 48 kDa and 45 kDa, respectively (Baranova et al., 2004). In contrast, Panx2 is localized to chromosome 22 and encodes a 70 kDa protein, making it the largest Panx family member characterized by an extended C-terminal domain (Baranova et al., 2004). Sequence analysis demonstrates limited structural conservation among Panx members, sharing only 50%–60% amino acid identity. However, structural conversation between different species is as high as 90% (Baranova et al., 2004). This evolutionary preservation underscores the functional importance of Panx proteins and validates their study in cross-species experimental models.

Panx proteins adopt a canonical four-transmembrane (4TM) topology, featuring two extracellular loops (ECL1/ECL2), cytoplasmic N- and C-termini, and an intracellular loop. Among the three family members, Panx1 has garnered the most attention, particularly for its ATP release during apoptotic cell clearance. ATP and UTP release (>80 nM) functions as a “find-me” signal that recruit phagocytes, enabling immunologically silent clearance of apoptotic cells (Chekeni et al., 2010; Elliott et al., 2009). Apoptotic stimuli trigger Panx1 channel activation through caspase3/7-mediated proteolytic cleavage at residues 376–379 of C-terminal domain (CTD) (Sandilos et al., 2012; Penuela et al., 2014a). This cleavage modification liberates a C-terminal autoinhibitory domain (CAD), relieving steric hindrance and inducing conformational changes in the N-terminal domain (NTD) to facilitate channel opening (Henze et al., 2024). Furthermore, hemichannels formed by truncated Panx1 (but not wild-type) permit Ca^2+^ influx that enables cell death without additional stimuli (Salgado et al., 2024). Importantly, functional analysis confirms that the Panx1 lacking NTD not only abolished functional channel opening but also exerted dominant-negative suppression of channel activity (Kuzuya et al., 2022). In addition, murine Panx2 was cleaved at aspartic acid (D) site Asp416 (equivalent to Asp415 of human Panx2) by caspase-3/7 activation (Sanchez-Pupo et al., 2022).

The ECL1/ECL2 of Panx1 constitute critical regulatory modules governing channel inhibition and ionic permeability (Michalski et al., 2020; Michalski and Kawate, 2016). Michalski and Kawate identified Tryptophan (W) site Trp74 in ECL1 as a key determinant of Panx1 sensitivity to classical inhibitors (CBX/PBN), with W74A mutations significantly attenuating drug-induced channel blockade (Michalski and Kawate, 2016). Subsequent investigations by the same team revealed that an inter-subunit cation-π interaction between Trp74 and Arg75 in ECL1 is essential for establishing Panx1’s ion selectivity profile (Michalski et al., 2020). And, substitutions at positions Ile247, Val258, and Phe262 in ECL2 were shown to selectively impair CBX-mediated inhibition (Michalski et al., 2020). These findings led to a hypothesis: rather than binding discrete amino acid motifs in ECL1, CBX exerts its inhibitory effect by stabilizing a closed-channel conformation through dynamic interactions bridging ECL1 and ECL2.

The ECLs serve as critical platforms for post-translational glycosylation that dictates subcellular trafficking of Panx proteins. Panx1 undergoes N-linked glycosylation at Asparagine (N) site Asn254 within ECL2, whereas Panx2 and Panx3 are modified at Asn86 (ECL1) and Asn71 within ECL1, respectively (Penuela et al., 2007; Sanchez-Pupo et al., 2018). Glycosylation is indispensable for plasma membrane localization of Panx1 and Panx3. Strikingly, Panx2 maintains constitutive localization to the endoplasmic reticulum (ER) and Golgi apparatus regardless of its glycosylation status (Penuela et al., 2007; Sanchez-Pupo et al., 2018).

Beyond glycosylation, Panx proteins undergo diverse post-translational modifications effecting channel gating and membrane trafficking, including phosphorylation (DeLalio et al., 2019; López et al., 2020; Medina et al., 2021; Metz and Elvers, 2022; López et al., 2021), deacetylation (Chiu et al., 2021), S-nitrosylation (Penuela et al., 2014a; Bunse et al., 2011; Lohman et al., 2012), ubiquitination and lipidation (D'Hondt et al., 2013). Lohman *et al* reported that S-nitrosylation of Panx1 via nitric oxide (NO) at cysteine (C) sites Cys40 and Cys346 inhibited Panx1 channel currents and ATP release (Lohman et al., 2012).

Panx1 channel could be phosphorylated at tyrosine (Y) sites Tyr150 (Nouri-Nejad et al., 2021), Tyr198 (DeLalio et al., 2019) and Tyr308 (Weilinger et al., 2012) via Sarcoma-family kinase (SFK) and at serine(S) site Ser205 via Salt-inducible kinase (SIK) (Medina et al., 2021), and at T302/S328 via Protein kinase A (PKA) (López et al., 2020), and at Ser394 via Ca^2+^/calmodulin-dependent kinase II (CaMKII) (López et al., 2021). Mutation of Tyr150 prevented glycosylation and trafficking of Panx1 protein (Nouri-Nejad et al., 2021). Tyr198, Tyr308 and Ser205 phosphorylation of Panx1 regulated channel activation. Immunostaining of Tyr198 phosphorylation was enriched in the smooth muscle layer of arteries from hypertensive humans, suggestive a role in hypertensive vascular pathology (DeLalio et al., 2019). Tyr308 phosphorylation was mediated by anoxia-induced NMDA receptor activation in pyramidal neurons (Weilinger et al., 2012). Expressing Ser205A mutated Panx1 mice phenocopied the exacerbated airway inflammation in Panx1 ablation mice (Medina et al., 2021). Mutation of either T302 or S328 prevented Panx1 channel activation induced by the mechanical stretch (López et al., 2020).

However, the authenticity of Panx1 phosphorylation modification has been challenged. Ruan et al reported that the commercially available antibodies against phosphorylation sites of Tyr198 and Tyr308 at Panx1 were non-specific (Ruan et al., 2024). This critical finding necessitates re-evaluation of prior phosphorylation studies reliant solely on immunodetection approaches.

### Tissue and cellular localization

The Panx family exhibits distinct tissue distribution patterns with important functional implications. Panx1, the most widely distributed member among the Panx proteins, is expressed in almost every organ of the human body (Baranova et al., 2004). Panx2 is expressed in the nervous system, thymus, heart, lung, stomach, and spleen (Baranova et al., 2004; Le Vasseur et al., 2014). While Panx3 is found in the skin, ear, mammary gland, testis, blood vessels, small intestine and skeletal system (O'Donnell and Penuela, 2021).

Panx proteins are widely distributed in various cell types, such as epithelial cells, endothelial cells, fibroblast, astrocytes, monocytes, blood cells, platelets, osteoblast, etc. The Panx channels functions related to diseases in different cell types have been summarized systematically in Tables 1, 2.

**TABLE 1:** Panx channels and inhibitors in inflammatory diseases pathogenesis and management.

I/R injury	Panx1 effect	Root cause cell type	Influencing factors	Panx1 inhibitor application	Administration method	References	Years
Lung	Promotion	Endothelial cell	IL-17, RANTES, CXCL1, MCP-1, TNFα and IL-6	CBX, PBN	*In Vivo*	29745255	2018
Kidney	Promotion	Endothelial cell, Epithelial cell	IL-6, Selectin E/P	CBX	*In Vivo*	29866797	2020
	Promotion	_	Caspase-11, NLRP3/IL-1β	CBX	*In Vitro*	33758356	2022
Brain	Promotion	Hippocampal neurons	Cathepsin B, calpian-1, Hsp70	PBN	*In Vivo*	26047730	2015
	Promotion	Endothelial celle	Leukocytes infiltration, cerebral myogenic tone	CBX, Spironolactone	*In Vitro*	29563335	2018
	Promotion	_	RIP3, HMGB1	10Panx	*In Vivo*	32173418	2020
Heart	Promotion	Cardiomyocytes	Mitochondrial respiration	_	_	37556386	2023

Other Inflammation	Panx1 Effect	Root Cause Cell Type	Influencing Factors	Panx1 Inhibitor Application	Administration Method	References	Years
Liver and lung injury	Promotion	_	Oxidative status	10Panx	*In Vivo*	27826632	2017
Liver fibrosis	Promotion	_	NF-kB/MIP1ɣ	_	*_*	29987408	2018
Acute Lung injury	Inhibition	Epithelial cell	Nras, Bcas2	_	*_*	35559667	2022
Acute Kidney injury	Promotion	_	NLRP3, IL-6, IL-1β, TNFα	CBX	*In Vivo*	32416166	2020
Asthma	Inhibition	CD4^+^ T cell	Adenosine	_	*_*	34283971	2021
Asthma	Promotion	_	_	CBX,10Panx	*In Vivo*	34231389	2021

Other Inflammation	Panx3 Effect	Root Cause Cell Type	Influencing Factors	Panx1 Inhibitor Application	Administration Method	References	Years
Osteoarthritis	Promotion	Chondrocyte	_	_		26138248	2015
Osteoarthritis	Inhibition	_	_	_		33426805	2021

**TABLE 2:** Panx channels and inhibitors in cancer pathogenesis and management.

Cancer type	Panx1 effect	Cell types	Function regulation	Mechanism	Inhibitor	Administration method	References
Breast Cancer	Promotion	Epithelial tumor cell	Microvascular metastasis	ATP-P2YR activation mediates cell survival	10Panx, CBX, PBN	10Panx, CBX, *in vivo* and *in vitro* PBN, *in vitro*	26098574
Breast Cancer	Promotion	Epithelial tumor cell	Metastasis	Regulated EMT pathway	PBN	*In Vitro*	31817827
Basal-like Breast Cancer	Promotion	_	Tumor immune microenvironment	Adenosine leads to neutrophil recruitment	_	_	35884429
Lung Adenocarcinoma	Promotion	Epithelial tumor cell	Drug resistance	P2Rs/iCa2+/PI3K/Akt/NF-κB/IL-6	_		37696858
Hepatocellular Carcinoma	Promotion	Epithelial tumor cell	Lung metastasis	Promoted EMT by AKT phosphorylation	_	_	31737105
Gastric Cancer	Promotion	Epithelial tumor cell	Metastasis	Affects EMT and AQP5 protein expression	_	_	34135208
Colorectal Cancer	Inhibition	Epithelial tumor cell	Cell survival	LXRs-PANX1/ATP-P2X7-NLRP3-caspase1	PBN, CBX	*In Vitro*	25124554
Colon Adenocarcinoma	Promotion	Epithelial tumor cell	Cell proliferation and tumorigenesis	_	10Panx, PBN	10Panx, PBN, *in vitro;* PBN, *in vivo*	38897345
Pancreatic ductal adenocarcinoma	Promotion	Epithelial tumor cell	Cell proliferation and tumorigenesis	UQCRC1-PANX1/ATP-P2Y2-PI3K/ATK	10Panx	*In Vitro* and *in vivo*	32089737
Pancreatic ductal adenocarcinoma	Promotion	Platelet	Cell migration, invasion and metastasis	PANX1/ATP/p38 MAPK/IL1β/EMT	10Panx, and PC63435*	10Panx, *in vitro,* PC63435 *in vivo*	36922676
Pancreatic ductal adenocarcinoma	Promotion	Epithelial tumor cell	cell proliferation and immunosuppression	NOD1-NF-kB-CCL2/PTGS2	_	_	39882247
Testicular Cancer	Promotion	Epithelial tumor cell	Cell migration and invasion	p-ERK1/2and MMP9	CBX, PBN	*In Vitro*	33345473
Testicular Cancer	Inhibition	Epithelial tumor cell	Drug resistance	ATP and IP3 inhibits cell apoptosis induced by DDP	_	*_*	28780469
Testicular Cancer	Inhibition	Epithelial tumor cell	Drug resistance	PANX1 via P-mTOR-p62 axis mediated autophagy	CBX, PBN	*In Vitro*	35373707
Melanoma	Promotion	Melanocyte	Cell proliferation, migration and invasion	Binds to N-terminal of beta-catenin	CBX, PBN	*In Vitro and* *in vivo*	30654593; 33647315
cutaneous Squamous Cell Carcinoma	Promotion	Squamous cell	Cell proliferation, migration	_	PBN, Spironolactone	*In Vitro*	39560179
Rhabdomyosarcoma	Inhibition	Rhabdomyoblastic cells	Cell proliferation and migration	Interact with AHNAK	_	*_*	30459312; 33564071
Glioma	Promotion	Astrocytes	Cell proliferation	Glutamic acid and IL-6,IL-8 released	_	_	25572468; 26385361
Tongue Cancer	Promotion	Microglia	Pain sensation	ATP/P2X7R/p-ERK	10Panx,	*In Vitro*	34768835

Cancer	Panx2 Effect	Cell Types	Function Regulation	Mechanmism	Panx1 Inhibitor Application	Administration Method	References
Colorectal Cancer	Promotion	Epithelial tumor cell	Cell proliferation, migration, tumorigenesis and drug resistance	PI3K/AKT and EMT	_	_	39389335
Prostate Cancer	Promotion	Epithelial tumor cell	Cell proliferation, migration and invasion	Associated with ferroptosis and NRF2 signaling pathway	_	_	32547072
Breast Cancer	Promotion	_	Metastasis	Regulated by oncRNA T3p,an oncRNA strongly promoted metastasis of breast cancer	_	_	30397354
Testicular cancer	Promotion	Epithelial tumor cell	Drug resistance	Affected expression of caspase-3, Bax and Bcl-2			32895173

Cancer	Panx3 Effect	Cell Types	Function Regulation	Mechanmism	Panx1 Inhibitor Application	Administration Method	References
cutaneous Squamous Cell Carcinoma	Inhibition	Squamous cell	Tumorigenesis	_	_	_	39560179
Osteosarcoma	Promotion	Spindle stromal cells	Cell proliferation, migration and invasion	Regulated by miR-431-5p	_	_	32982413

*PC63435 is a chemical compound composed of CBX and PSGL-1, specifically binds to CD62P+ platelet.

While Panx proteins were historically characterized as plasma membrane-localized hemi-channels mediating autocrine/paracrine communication (Penuela et al., 2007; Penuela et al., 2009), emerging studies have shown their localization on organelle membranes. Panx1 located in cardiac mitochondria and modulated cardiac susceptibility to ischemia-reperfusion injury (Rusiecka et al., 2023). Panx2 was observed in ER and Golgi apparatus (Sanchez-Pupo et al., 2018), and the contact sites of ER and mitochondria, sensitizing cells to apoptotic stimuli (Le Vasseur et al., 2019). Panx3 was identified in ER, regulating osteoblast differentiation (Ishikawa et al., 2011), and Golgi apparatus, regulating vascular oxidative stress in endothelial cells (Wolpe et al., 2024).

### Panx channels physiological function

Recent breakthroughs in Cryo-Electron microscopy (Cryo-EM) have resolved the heptameric architecture of Panx channels (Qu et al., 2020; Jin et al., 2020; Zhang et al., 2023; Tsuyama et al., 2025), revealing seven identical subunits arranged around a central pore with diameter constraints permitting selective permeation of molecules up to 1.5 kDa, including amino acids (Lazutkaite et al., 2017), nucleotides (Bao et al., 2004), and ions (Ma et al., 2012). The physiological functions of Panx channels in many organs have been well reviewed., including brain, skin, kidney, lung, heart, bladder, skeletal muscle, and digestive system, including liver and pancreas etc (Giaume et al., 2021; O'Donnell and Penuela, 2023; Abed et al., 2015; Badaoui and Chanson, 2023; Li et al., 2015; Andersson, 2015; Maes et al., 2015a; Maes et al., 2015b; Maes et al., 2014; Cigliola et al., 2015; Riquelme et al., 2013).

However, some details and new research contents still need to be supplemented here. Panx1 channel is also involved in the development of lactational mammary gland (Stewart et al., 2016) and oocyte (Kordowitzki et al., 2021). In pancreatic islet cells, Panx1 was found to be expressed in β and δ cells, and Panx2 in β, α, and δ cells, while Panx3 was absent in pancreas (Scemes et al., 2008; Berchtold et al., 2017; Miranda et al., 2022). Functional studies in INS-1E β -cells demonstrated Panx1 and Purinergic Receptor P2X 7 (P2X7) coupling drives glucose-induced ATP release, stimulating Ca^2+^ transients, cell proliferation and insulin secretion (Tozzi et al., 2018). Panx2 deficiency rendered pancreatic β cells susceptible to cytokine-induced apoptosis *in vitro* through hyperactivation of iNOS/STATs pathways and impaired glucose tolerance *in vivo* (Berchtold et al., 2017). Latest research shows that Panx2 deficiency can disrupt visual pathways and cause eye defects in zebrafish (Shanbhag et al., 2025). Panx3 primarily participates in physiological processes including cartilage/bone development, remodeling, and regeneration, as well as pathological conditions such as obesity, osteoarthritis, and degenerative disc disease (O'Donnell and Penuela, 2021; Ishikawa and Yamada, 2017). Emerging evidence reveals that Panx3 collaborates with the Purinergic Receptor P2X 4 (P2RX4) channel to sustain serum antibody titers and promote the survival of bone marrow plasma cells (Ishikawa et al., 2024).

Accumulating data indicate potential functional redundancy may exist among the Panx isoforms. In the skin, the expression level of the Panx3 was significantly upregulated in Panx1 KO mice (Penuela et al., 2014b). Additionally, both Panx1 and Panx3 are capable of recruiting monocytes (Chekeni et al., 2010; Pillon et al., 2014). A study on cerebral ischemic stroke revealed that Panx1 gene KO had no effect on the disease, while dual Panx1 and Panx2 ablation significantly alleviated the obstruction phenomenon (Bargiotas et al., 2011). In cutaneous squamous cell carcinoma (cSCC), Panx1 and Panx3 exhibit divergent tumor-modulatory roles. Panx1 drives tumor progression through ubiquitous overexpression that accelerates malignant proliferation and invasion, whereas Panx3 exerts tumor-suppressive effects (O'Donnell, 2024).

## Panx channels in pathological processes

In various pathological processes, especially in inflammation and tumorigenesis, the expression level and channel open status of Panx channels undergoes significant changes. Understanding the patterns and underlying mechanisms of these changes in these processes possibly helps us better diagnose and treat patients.

### Panx channels in ischemia-reperfusion injury and other inflammation

#### Ischemia-reperfusion injury

Ischemia-reperfusion (I/R) injury represents a clinically significant pathological process wherein reperfusion paradoxically exacerbates tissue damage through amplified inflammation and reactive oxygen species (ROS) generation, ultimately driving apoptotic, autophagic, and necrotic cell death cascades (Wu et al., 2018).

Over the past decade, Panx1 has emerged as a central mediator of I/R injury across multiple organ systems, including the brain (Good et al., 2018a), heart (Rusiecka et al., 2023), lungs (Sharma et al., 2018), and kidneys (Jankowski et al., 2018; Yi et al., 2022).

Mechanistic studies delineate its endothelial-specific pathogenesis. In cerebral I/R injury, endothelial Panx1 ablation, rather than smooth muscle Panx1, significantly reduced infarct volume, suppressed leukocyte infiltration and normalized cerebral vascular tone (Good et al., 2018a). In pulmonary I/R injury, endothelial Panx1 KO prevented vascular hyperpermeability, attenuated edema formation and preserved lung compliance by blocking neutrophils infiltration (Sharma et al., 2018). In renal I/R injury, tissue-specific Panx1 deletion in proximal tubules and endothelia protected kidneys from IRI, correlating with reducing cytokine, chemokine, and leukocyte adhesion molecules expression (Jankowski et al., 2018). Mechanistically, a separate study demonstrated that hypoxia/reoxygenation markedly upregulates caspase-11 expression in cultured primary tubular cells, leading to Panx1 cleavage. This proteolytic event triggers NLRP3 inflammasome activation, driving inflammation and renal injury in I/R-induced acute kidney injury (AKI) (Yi et al., 2022).

Notably, cardiomyocyte Panx1 deficiency disrupted mitochondrial ATP efflux, resulting in accumulation of ATP that attenuated cardiac tissue sensitivity to I/R-induced metabolic crisis (Rusiecka et al., 2023).

#### Other inflammation

Panx1 exhibits duality in allergic asthma pathogenesis. Firstly, Panx1 inhibited asthma pathogenesis by facilitating immune tolerance of Treg-Teff cell crosstalk via adenosine signaling (Medina et al., 2021). Clinical data reveal decreased Panx1 mRNA in peripheral blood mononuclear cells (PBMC) of pediatric asthma patients, while CD4^+^ T cell-specific Panx1 KO mice showed exacerbated House Dust Mite (HDM)-induced airway inflammation reversible upon Panx1 reconstitution (Medina et al., 2021). Mechanistically, ATP released by Regulatory T cells (Treg) and T-effector cells (Teff) hydrolyzed to adenosine via ectonucleoside triphosphate diphosphohydrolase-1 (ENTPD1/CD39) and Ecto-5′-nucleotidase (NT5E/CD73) could inhibit excessive proliferation of Teff cells. Unlike ATP, adenosine is an anti-inflammatory factor (Antonioli et al., 2013). Conversely, Panx1 potentiated asthma pathogenesis by enabling extracellular ATP accumulation in tracheal fluid that impaired mucociliary clearance (Arzola-Martínez et al., 2021). And Panx1 channel inhibition reduced extracellular ATP levels and airway hyperreactivity (AHR) in the allergic mouse model.

Panx1 orchestrates tissue repair processes through fibrosis initiation, demonstrating dual regulatory roles in wound healing pathophysiology. As a fundamental pathophysiological process, tissue damage triggers cytokine-mediated inflammatory cascades that activate both regenerative and fibrotic responses to maintain structural integrity (Oishi and Manabe, 2018). While controlled fibrosis preserves organ architecture, excessive extracellular matrix deposition culminates in functional impairment. When lung and liver are injured, endothelial cells released alarming cytokine interleukin 1 alpha (IL-1α) to serve as first line mediator to initiate repair response. Receptor-interacting protein kinase 1(RIPK1) regulated the transcription of IL-1α and caspase-8 promotes the cleavage of IL-1α. Panx1 channel on vascular endothelial cells mediated the secretion of cleaved IL-1α into extracellular to initiate organ fibrosis (Zhang C. et al., 2024). In contrast, bile duct ligation model reveals Panx1’s protective hepatic role. In bile ligation induced liver fibrosis, Panx1 mRNA expression level was increased and Panx1-KO mice exhibited exacerbated liver damage, increased oxidative stress and elevated numbers of macrophages recruitment (Crespo Yanguas et al., 2018).

Furthermore, Panx1 is implicated in chronic pain. Global or neuron-specific Panx1 deletion markedly decreased pain thresholds after complete Freund’s adjuvant (CFA) stimuli, indicating that the essential role of Panx1 in maintaining pain sensitization (Xing et al., 2024). Neuronal Panx1 ablation also markedly reduced differentiation in cultured neurons, hindered activation of Wnt/β-catenin signaling, diminished cell excitability and response to ATP stimulation (Xing et al., 2024).

Panx3 is suggested to be associated with osteoarthritis. Panx3 immunostaining was increased in areas of cartilage degeneration in mice and humans. Cartilage-specific or global deletion of Panx3 protected against development of osteoarthritis induced by destabilization of medial meniscus (DMM) surgery (Moon et al., 2015). However, they also reported that systemic Panx3 deletion led to accelerated progression of aging-induced osteoarthritis in mice (Moon et al., 2021). It can be seen that Panx3 plays opposite roles in osteoarthritis induced by different factors, and the mechanism is still unknown.

### Panx channels in tumorigenesis

Emerging evidence delineates the functional pleiotropy of Panx channels in tumorigenesis, with isoforms-specific roles spanning tumor-intrinsic programs.

#### Panx channels as tumor prognostic indicators

Clinically, Panx expression signatures serve as potent prognostic indicators. Elevated Panx1 expression predicts poor clinical outcomes in specific contexts. Bulk RNA sequencing data showed that the mRNA expression of Panx1 was significantly upregulated in the vast majority tumors in the Oncomine database, including breast cancer, cervical cancer, colorectal cancer, esophageal cancer, gastric cancer, head and neck cancer, kidney cancer, lymphoma, leukemia, lung cancer, pancreatic cancer, and sarcoma. Among them, high expression of Panx1 was significantly associated with shorter overall survival or disease-free survival in patients with kidney, lung, pancreatic, and endometrial cancer (Bao et al., 2021). In addition, extra reports suggest that Panx1 is highly expressed in breast cancer (Stewart et al., 2016), colon cancer (Fierro-Arenas et al., 2024), and cSCC (O'Donnell, 2024), and is associated with poor prognosis in patients.

Panx2 was highly expressed in tumor tissues of colorectal cancer (Zhang K. et al., 2024), bladder cancer (Liao et al., 2020), clear cell renal cell carcinoma (ccRCC) (Kim et al., 2019), breast cancer (Fish et al., 2018; Qian et al., 2021), esophageal cancer (Zhang D. et al., 2021), and cholangiocarcinoma (Liu et al., 2019), and its highly expression was significantly associated with the poor prognosis of patients. And Panx2, together with hsa-miR-105-5p and BCAR1, was identified as a reliable biomarker related to breast cancer metastasis (Qian et al., 2021). Contrastingly, Panx2 could also function as a potential tumor suppressor gene. In glioma, as well as brain low grade glioma, Panx2 expression was reduced, and its low expression predicted worse patient survival (Lai et al., 2009; Xu et al., 2021). Panx2 was found to have a high degree of DNA methylation in Myelodysplastic Syndrome (MDS) (Zhou et al., 2022) and hepatocellular carcinoma (HCC) (Xie et al., 2015).

Panx3 was highly expressed in osteosarcoma and exerted pro-cancer function (Sun S. et al., 2020; Poudel and Koks, 2024). Meanwhile, as one of the arachidonic acid metabolism related genes (AAMRGs), CD36, CLDN11, EPYC, Panx3 and STOM, high expression of AAMRGs is significantly associated with poor prognosis of osteosarcoma (W et al., 2025). Recently, Panx3 and Glioma-associated homologue-1 (GLI1) gene fusions and MDM2 amplification are also found in a case of epithelioid neoplasm (ES) in a 13-year-old child, suggesting a genetic correlation between Panx3 mutations and ES (Konovalov et al., 2025).

#### Regulating tumor cell proliferation

Panx1 is crucial for tumor cell proliferation, including melanoma, cSCC, and pancreatic cancer (O'Donnell, 2024; Freeman et al., 2019; Sayedyahossein et al., 2021; Wang et al., 2020; Wu et al., 2024). Paradoxically, Panx1 exhibits tumor-suppressive functions in rhabdomyosarcoma (Xiang et al., 2018; Xiang et al., 2021). And Panx1 have both promoting and restricting tumor functions in colon cancer (Fierro-Arenas et al., 2024; Derangère et al., 2014) and malignant glioma cells (Lai et al., 2007; Wei L. et al., 2015; Wei et al., 2016).

In melanoma, Panx1 interacts with β-catenin through its N-terminus, contributing to cancer cell proliferation and tumor formation (Freeman et al., 2019; Sayedyahossein et al., 2021). In cSCC, Panx1 promotes SCC-13 cells growth and migration (O'Donnell, 2024). In PDAC, as down-regulator of mitochondrial protein Ubiquinol-Cytochrome C Reductase Core Protein 1 (UQCRC1), Panx1 releases ATP extracellularly, which functions on Purinergic Receptor P2Y 2 (P2Y2) via autocrine action, activating RTK-PI3K-AKT signaling pathway and thus promoting cancer cell proliferation (Wang et al., 2020). P2Y2 is one of G-protein-coupled receptors (GPCR) and is a receptor for ATP and UTP (Linden et al., 2019). In PDAC, high P2RY2 expression correlates with poor prognosis in patients. Mechanically, ATP-activates P2Y2 receptors engaged in cross-talk with Platelet-Derived Growth Factor Receptor (PDGFR) via Yes1-mediated transactivation, initiating PI3K/AKT-mTOR signaling and upregulating c-Myc and HIF1-α. This transcriptional reprogramming drives cancer cell glycolysis (Hu et al., 2019). In addition, recent report shows that Panx1 overexpression can facilitates tumor growth in Panc02, a mice PDAC cell line (Wu et al., 2024). Furthermore, in platelets, Panx1 released ATP influencing the expression of phosphorylated p38/MAPK and NOD-like receptor thermal protein domain associated protein 3 (NLRP3) via purinergic receptor P2X 1 (P2RX1) and the release of Interleukin 1 beta (IL-1β), acting on PDAC cells could promote the EMT process, facilitating their invasion and metastasis (Li et al., 2023).

In rhabdomyosarcoma, Panx1 impeded tumor cell proliferation due to interaction with bracket proteins, which stabling cell structure (Xiang et al., 2018; Xiang et al., 2021). In colorectal cancer, Panx1 was involved in the Liver X receptor β (LXRβ) activation-mediated Panx1-P2X7-NLRP3-Caspase1 axis-dependent pyroptosis of tumor cells, performing anti-cancer function (Derangère et al., 2014). However, recent study has shown that Panx1 is significantly elevated in colorectal cancer, and inhibiting Panx1 can obstruct tumor cell proliferation and tumor growth (Fierro-Arenas et al., 2024). Overexpression of Panx1 in malignant glioma cells hindered C6 cell proliferation [17308093]. Conversely, Panx1 in U87MG cell influenced the release of glutamate and cytokines IL-6 and IL-8, acting anti-tumor effect (Wei L. et al., 2015; Wei et al., 2016). Different cell lines of malignant glioma yield opposite results.

Panx2 serves as tumor promotion factor in colorectal cancer (Zhang K. et al., 2024) and prostate cancer (Liao et al., 2020), while Panx2 performs anti-tumor function in glioma (Lai et al., 2009).

In colorectal cancer, Panx2 promoted tumor cell proliferation, migration, tumorigenesis and drug resistance through PI3K/AKT and epithelial-mesenchymal transition (EMT) signaling pathway (Zhang K. et al., 2024). In prostate cancer, Panx2 facilitated tumor cell proliferation, migration and invasion via ferroptosis and Nuclear Factor Erythroid 2-Related Factor 2 (NRF2) signaling pathway (Liao et al., 2020). In addition, in ccRCC, FAM83H significantly preferred cell proliferation, and Panx2 expression was regulated by FAM83H, suggesting that Panx2 may related to cell proliferation (Kim et al., 2019). In rat C6 glioma cells, the expression of Panx2 was significantly reduced, and restoring its expression could inhibit cell proliferation and tumor growth *in vivo*, indicating the tumor suppressor effect of Panx2 (Lai et al., 2009).

Highly expressed Panx3 inhibited apoptosis and promoted the proliferation, migration and invasion of osteosarcoma cells. MicroRNA-431-5p could target and regulate the expression of Panx3, exerting tumor suppressor function (Sun S. et al., 2020). In addition, Panx3 could hinder osteoprogenitor cell growth by promoting the degradation of β-catenin and inhibiting transcription of cell cyclin related genes (Ishikawa et al., 2014). In cSCC, Panx3 exhibits tumor suppressive properties. Panx3 transcripts were reduced, and Panx3 systemic knockout mice could be more susceptible to precancerous papillomas (O'Donnell, 2024).

#### Regulating tumor cell migration, invasive and metastasis

Panx1 promotes tumor metastatic progression in multiple epithelial-derived tumors including breast, gastric, hepatocellular, testicular, melanoma, and cSCC (O'Donnell, 2024; Freeman et al., 2019; Sayedyahossein et al., 2021; Furlow et al., 2015; Jalaleddine et al., 2019; Ying et al., 2021; Shi et al., 2019). Paradoxically, in mesenchymal-origin rhabdomyosarcoma (Xiang et al., 2021), Panx1 reduced cell viability and migration through combination with the AHNAK scaffold protein in the pseudopodal protrusion.

The metastatic cascade imposes mechanical stress on circulating tumor cells, rendering them susceptible to deformation-induced apoptosis during intravascular transit (Wong et al., 2001; Kienast et al., 2010). Intriguingly, metastatic breast cancer cells evolved survival strategies through Panx1 truncation mutants (1-89aa), which exhibited force-dependent ATP efflux. This mechano-activated purinergic signaling engaged P2Y receptors to suppress caspase-3/7 cleavage mediated intravascular cell death, thereby conferring anoikis resistance essential for metastatic colonization (Furlow et al., 2015).

In addition, Panx1 regulates tumor cell migration and invasion mainly by EMT signaling. Panx1 was regulated by the aquaporin5 (AQP5), promoting the migration, invasion and EMT of gastric cancer cells (Ying et al., 2021). Panx1 regulated the cell migration and invasion in hepatocarcinoma, and depended on the Protein Kinase B (PKB/AKT) signaling pathway to promote EMT progression (Shi et al., 2019). In testicular cancer, inhibition Panx1 function hindered cell migration and invasion, and downregulated the expression of p-ERK1/2, vimentin and matrix metalloproteinase 9 (MMP9) (Shi et al., 2019).

Emerging evidence positions Panx2 as a pan-cancer metastasis regulator through distinct molecular architectures. Expression of Panx2 was significantly correlated with tumor cell migration in colorectal cancer (Zhang K. et al., 2024) and with metastasis in breast cancer (Fish et al., 2018). Panx2 expression was regulated by Orphan non-coding RNA T3p (oncRNA T3p), a powerful promoter of breast cancer metastasis, suggesting a promoting metastasis function of Panx2 (Fish et al., 2018).

#### Regulating tumor cell drug resistance

Cisplatin (DDP) is a platinum-based chemotherapy drug that triggers apoptosis in cancer cells by blocking DNA replication and transcription through cross-linked DNA double-strands. However, some of tumor cells develop DDP resistance by enhancing DNA repair, activating anti-apoptotic pathways (e.g., Bcl-2 family proteins associated apoptosis), or reducing drug accumulation. Tong et al suggested that high Panx1 expression resulted in testicular cancer cells more sensitive to the killing effect of DDP. The lower expression of Panx1 in DDP-resistant cell line, I-10/DDP cells, led to a simultaneous decrease in the content of extracellular ATP and intracellular inositol triphosphate (IP3) and consistent with inhibiting DDP-induced apoptosis (Wu et al., 2017). In addition, they also uncovered that in testicular cancer cells, overexpression of Panx1 was consistent with Mechanistic Target of Rapamycin (mTOR) phosphorylated and higher p62 levels, inhibiting autophagy caused by DDP resistance (Yuan et al., 2022). These results argued that patients with testicular cancer with high expression of Panx1 might be more sensitive to DNA-damaging chemotherapeutic agents. In addition, they proposed that Panx2 protein levels were higher in testicular cancer-resistant cell lines, and downregulation of Panx2 led to decreased caspase-3 and BCL2-associated X protein (BAX) expression, and increased B-cell lymphoma 2 (BCL2) expression, enhanced DDP-induced apoptosis in testicular cancer I-10 cells (Yao et al., 2020), which argued that patients with low Panx2 expression might be more sensitive to the therapeutic effect of DDP.

In colorectal cancer, the Panx2 high expression group showed a significantly higher half maximal inhibitory concentration (IC50) value for 5-Fluorouracil (5-FU), a commonly used chemotherapeutic drug for colorectal cancer. And overexpressed Panx2 could prevent tumor cells from killing by 5-FU (Zhang K. et al., 2024). Suggesting treatment of Panx2 inhibitors combined with 5-FU can enhance the therapeutic effect of 5-FU single used on colorectal cancer.

In conclusion, high Panx1 expression could sensitize patients with testicular cancer to chemotherapy drug DDP treatment. However, high Panx2 expression in testicular and colorectal cancer could help tumor cells survival and growth facing DDP or 5-FU treatment. These results may serve as clinical medication indicators, guiding doctors in the clinical treatment of testicular cancer and oral cancer patients.

## Panx channels in immune response

Immune responses are physiological defense reactions against exogenous factors, composed of innate and adaptive response (Chaplin, 2010). The innate response provides rapid, nonspecific defense via physical barriers, phagocytes (neutrophils, macrophages), and soluble factors (cytokines, complement). Conversely, the adaptive response is delayed but antigen-specific, mediated by T cells, B cells, dendritic cells, and antibodies (immunoglobulins) targeting specific antigens.

Igor Santiago-Carvalho et al. systematically reviewed that Panxs are crucial regulators of adaptive immune response in disease contexts such as cancer or viral infections (Santiago-Carvalho et al., 2024). Briefly, Panx1 modulates T cell development, initial activation, effector function, and memory responses. Panx3 regulates bone development to promote humoral immunity, whereas the role of Panx2 in immune responses remains incompletely defined.

Here, we conclude the effects of Panx channels on the immune response in infectious and tumoral diseases.

### Panx channels in infectious diseases

Panx1 channel, particularly in endothelial cells, proximal tubules, and cardiomyocytes, promotes sterile inflammation by releasing ATP. This drives cytokine/chemokine expression, NLRP3 inflammasome activation, and neutrophil infiltration across diverse ischemic and nonischemic organ injuries (Sharma et al., 2018; Jankowski et al., 2018; Yi et al., 2022; Pavelec et al., 2024). During fibrosis, Panx1 promotes macrophage recruitment (Crespo Yanguas et al., 2018). In allergic responses, Panx1 primarily mediates extracellular adenosine accumulation, thereby influencing adaptive immunity by regulating Treg-Teff cell crosstalk (Medina et al., 2021).

Inhibiting Panx1 activity or knock down its expression suppresses expression of proinflammatory cytokine, chemokine and leukocyte adhesion molecule while reducing neutrophils infiltration or macrophage recruitment in ischemic and nonischemic organ injuries, conversely, it aggravates allergy severity.

### Panx channels in tumor microenvironment

Panx1 is proposing as a valid immune-related therapeutic target for cancer due to regulating immune cells infiltration especially neutrophils. Tumor associated neutrophils and macrophages are abundantly in tumor-immune microenvironment serve as immunosuppressive functions (Kumar et al., 2016). Neutrophils release their chromatin extracellular and assemble into web-like structural neutrophil extracellular Traps (NETs). NETs involve in immune protection, inflammatory (Papayannopoulos, 2018) and auto-immune diseases (Lee et al., 2017) and cancer (Adrover et al., 2023).

Based on the analysis of whole transcriptome sequencing data, the expression level of Panx1 was related to the immune infiltration, affecting the infiltration of neutrophils, tumor-associated fibroblasts, macrophages, myeloid-derived dendritic cells (MDSCs), and monocytes in PDAC (Bao et al., 2021). Analysis of bulk RNA sequencing data in breast cancer indicated that Panx1 was highly expressed in tissue of basal-like breast cancer, positively correlating with the expression of ENTPD1 (CD39)/NT5E (CD73) in the tumor microenvironment, and showing a significant correlation with tumor-associated neutrophils infiltration (Chen et al., 2022). Mukai et al. demonstrated that Spermidine released by Panx1 channel in apoptotic tumor cells could mediate a new immune-escape mechanism via inducing neutrophil extracellular traps (NETs) and shaping the immune rejection microenvironment (Mukai et al., 2022). Damage Associated Molecular Patterns (DAMPs) refer to endogenous molecules released by dead cells, which are endogenous danger signals. A risk assessment model for PDAC immunotherapy based on DAMPs molecules found that PDAC patients with high DAMPs expression had worse prognosis and immune status. Panx1 is one of the DAMPs molecules, activating the NOD1/NF-κB signaling pathway through ATP release, promoting tumor progression and immune regulation (Wu et al., 2024). In Low-grade glioma (LGG), the expression of Panx2 was negatively correlated with the infiltrating levels of macrophages, dendritic cells, and CD4 T cells (Xu et al., 2021).

Notably, whole-transcriptome analyses identified Panx1 as a ferroptosis- associated genes whose dysregulation correlated with drug resistance, tumor immune infiltration, and tumor stemness in lung adenocarcinoma (LUAD) (Zhang A. et al., 2021; Ren et al., 2021). In LUAD, Panx1 on cancer cells was transcriptionally regulated by p53 protein and assisted to IL-6 production and secretion via ATP/P2 receptors/Ca2+/AKT/NF-κB signaling axis. Secreted IL-6 could promote the polarization of macrophages into M2-like phenotype which enhancing pro-metastatic effects on cancer cells (Phan et al., 2023).

## Treatment of Panx1 channel inhibitor in diseases

### Panx1 channel inhibitors

As previously mentioned, Panx channels are involved in inflammatory and cancer processes and playing important functions. Clinical suppression of Panx channels function may be able to assist in disease treatment. Currently, Panx channel inhibitors mainly target the Panx1 channel, and there are no reported inhibitors for Panx2 or Panx3, which is a significant issue that needs to be addressed in future. Koval *et al* systematically reviewed Panx1 channel blocking reagents, focusing on chemical reagents and small molecular mimetic peptides. Additionally, for clinical drugs, they compared the advantages, the specificity and feasibility of clinical applications (Koval et al., 2023).

At present, the commonly used Panx1 inhibitors for researchers are Probenecid (PBN), Carbenoxolone (CBX), and 10Panx. Firstly, PBN is an FDA-approved drug for the treatment of gout and has been found to be one of the effective drugs blocking Panx1 channel (Silverman et al., 2008). It is also reported as an inhibitor of Urate Transporter 1(URAT1/SLC22A11) (Shin et al., 2011; Ebert et al., 2014), ABCC4/MRP4 (Ventimiglia et al., 2015), Organic Anion Transporters(OATs) (Granados et al., 2022), Organic cation transporters (OCTs) and P2X7 (García-R et al., 2023). Secondly, CBX, historically utilized for gastric ulcer treatment (Henman, 1970), is currently serves as a broad-spectrum inhibitor of both Panx and Connexin channels (Davidson et al., 1986; Bruzzone et al., 2005). Thirdly, 10Panx is a 10-peptide fragment targeting the W74 to Y83 segment of the ECL1 of Panx1 protein, capable of specifically inhibiting Panx1 function (Pelegrin and Surprenant, 2006). 10Panx is a good study tool for the Panx channel function research, but it cannot be used for clinical treatment. Finally, Spironolactone is an effective anti-hypertensive drug and is also one of the inhibitors of Panx1 channel (Good et al., 2018b). Critically, all known pharmacological blockers of Panx1 channels lack selectivity, necessitating the development of novel targeted inhibitors. Moreover, current Panx1 blockers require high concentrations *in vitro* or doses *in vivo* to achieve efficacy, which may elicit significant off-target effects (Koval et al., 2023). Particularly, CBX is excluded from contemporary first-line regimens for gastric ulcer management (Kavitt et al., 2019).

### Panx1 channel inhibitor in inflammation

Panx1 channel inhibitors play a significant role in the treatment of brain, liver, lung, and kidney injuries. Firstly, following brain ischemia, PBN pretreatment could inhibit reperfusion induced CA1 neuron death. Furthermore, administering PBN continuously for 7 days after reperfusion protected nerve cells from I/R damage (Wei R. et al., 2015). *In vitro* treatment of isolated posterior cerebral arteries with CBX or Spironolactone significantly dampened the development of brain muscle-derived muscle tone, similar to the phenomenon that application with the Apyrase, an ATP hydrolyzing enzyme (Good et al., 2018a). *In vivo* administration of 10Panx reduced the volume of brain infarction and alleviated neurological deficits as well as vascular inflammatory damage (Wei et al., 2020).

Secondly, administration of 10Panx *in vivo* inhibited liver damage caused by excessive use of acetaminophen (APAP), suppressed the recruitment of neutrophils by reducing Panx1 function, improved oxidative state of liver, and promoted tissue regeneration (Maes et al., 2017).

Thirdly, in mice asthma induced by OVA, ATP released by Panx1 worse inflammatory response. Inhibiting Panx1 function with 10Panx reduced airway hyperreactivity in lung (Arzola-Martínez et al., 2021). In addition, pretreatment of CBX or PBN *in vivo* protected lung tissue from I/R-induced tissue edema, vascular permeability changes, inflammatory factor release, as well as neutrophils infiltration and activation (Sharma et al., 2018).

Fourth, CBX pretreatment protected kidney from I/R injury (Jankowski et al., 2018). Furthermore, Fan Yin et al. supposed Panx1 facilitated acute kidney injury (AKI) via ATP/NLRP3/Caspase11/IL1β signaling axis (Yi et al., 2022). In another research, CBX inhibited Panx1-mediated NLRP3 activation and the release of IL-6, TNF-α, and IL-1β, contributing to the inhibition of renal sepsis injury (Huang et al., 2020).

### Panx channels inhibitor in cancer

The application of CBX, PBN, or 10Panx *in vitro* or *in vivo* can inhibit the proliferation, migration, invasion, and tumor formation of cancer cells, and even alter their sensitivity to chemotherapy drugs. Firstly, in colorectal cancer, *in vitro* experiments showed that PBN and 10Panx significantly inhibited the proliferation of colon cancer cells. *In vivo* experiments indicated that intraperitoneal injection of PBN in mice promoted tumor necrosis and inhibited subcutaneous tumor proliferation (Fierro-Arenas et al., 2024). In PDAC, intratumoral injection 10Panx inhibited UQCRC1 overexpressed tumor growth (Wang et al., 2020).

The application of 10Panx *in vitro* inhibited the function of Panx1 in platelets, and its culture medium hindered tumor growth, migration, and invasion of co-cultured PDAC cells. In addition, the P-selectin glycoprotein ligand-1 (PSGL-1) is a chemical molecule specifically band to the CD62P on the platelets. Chemically synthesized PSGL-1 with CBX produced PC63435, a specific Panx1 inhibitor band to platelets, blocked the Panx1/IL-1β pathway, suppressed PDAC tumor invasion and metastasis *in vivo* (Li et al., 2023).

In breast cancer, the truncated form of Panx1 (1–89) promoted the survival of metastatic breast cancer cells and retained sensitivity to CBX, 10Panx, and PBN (Furlow et al., 2015). PBN also blocked the Panx1-mediated EMT signaling pathway, preventing the invasive and metastatic characteristics of breast cancer cells (Jalaleddine et al., 2019). CBX and PBN reduced the invasion and migration capabilities of testicular cancer Tcam-2 cells (Sun Y. Y. et al., 2020) and inhibited cancer cell growth and migration in melanoma (Freeman et al., 2019). In squamous cell carcinoma, PBN and Spironolactone attenuated cell proliferation and migration (O'Donnell, 2024).

In addition, CBX and PBN preferred autophagy in the I-10/DDP drug-resistant cell line of testicular cancer, indicating that blocking Panx1 channel function sensitized tumor cells to DPP treatment (Yuan et al., 2022).

## Conclusion and future perspectives

Panx channels are localized to the cellular membrane and regulate releasing signaling molecules (e.g., ATP), metabolites and ions, modulating diverse physiological and pathological processes. In the processes of inflammation and cancer, Panx channels are involved in mediating inflammatory cell recruitment, cytokine secretion cascades, tumor cell proliferation/metastasis, and immune microenvironment remodeling. Many application-based studies on Panx1 channels have demonstrated the therapeutic potential of pharmacological Panx1 inhibition in attenuating disease progression across preclinical models. However, before applying Panx inhibitors in clinical treatments for inflammation or cancer, we still have many studies that need to be conducted.

**FIGURE 2:**
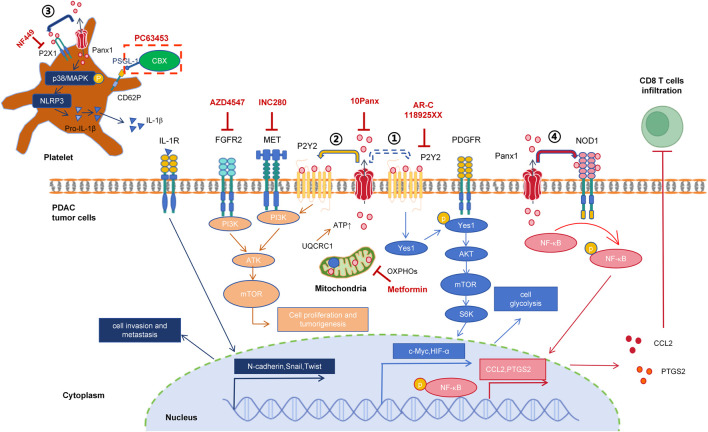
Mechanistic insights into Panx1-driven oncogenic signaling in pancreatic ductal adenocarcinoma. ① 2019: Metabolic reprogramming (Hu et al., 2019). ATP (potentially Panx1-independent, dashed arrow) activates P2RY2, inducing Yes1-mediated PDGFR transactivation. This triggers PI3K/AKT/mTOR/S6K signaling, upregulating c-Myc and HIF1α to enhance glycolysis. Inhibitors: AR-C118925XX (P2RY2 antagonist) + gemcitabine block tumor progression. ② 2020: Mitochondrial-ATP axis (Wang et al., 2020). UQCRC1 sustains OXPHOS to fuel Panx1-dependent ATP release. Extracellular ATP activates P2RY2, which cross-talks with FGFR2/MET to drive AKT phosphorylation, promoting proliferation and tumorgenesis. Inhibitors: Metformin (OXPHOS blocker), 10Panx (Panx1 blocker), AZD4547 (FGFR2 antagonist), INC280 (c-MET inhibitor), AR-C118925XX. ③ 2023: Platelet-mediated metastasis (Li et al., 2023). Platelet Panx1 releases ATP to activate P2X1, inducing p38/MAPK phosphorylation and NLRP3/IL-1β release. IL-1β promotes EMT in PDAC cells. Inhibitors: PC63435 (CBX-PSGL-1 conjugate targeting CD62P^+^ platelets) suppresses invasion and metastasis. ④ 2025: Immune suppression (Wu et al., 2024). Panx1 activates NOD1/NF-κB signaling, increasing CCL2 and PTGS2 expression while inhibiting CD8^+^ T-cell infiltration.

Taking PDAC treatment as an example. PDAC is a highly lethal malignant tumor and has become the third leading cause of cancer death in the United States (Siegel et al., 2024). Due to its hidden clinical symptoms, nearly half of PDAC patients are diagnosed at the distant metastatic stage, with a survival rate of only 3% (Siegel et al., 2020). Bioinformatic analysis and predictions have identified that high expression of Panx1 was significantly associated with shorter survival time in PDAC patients and worse immune infiltration (Bao et al., 2021). PDAC is generally divided into exocrine and endocrine types, with about 90% of clinical PDAC patients presenting as PDAC. Experimental results indicate that high expression of Panx1 in PDAC cells promotes tumor growth (Wang et al., 2020; Wu et al., 2024). And high expression of Panx1 in platelets facilitates the invasion and metastasis of PDAC cells (Li et al., 2023). 10Panx suppresses PDAC cell tumor growth mediated by mitochondrial protein UQCRC1 (Wang et al., 2020), as well as prevents the pro-migratory and invasive effects of platelets on PDAC cells (Li et al., 2023). Integrative analysis of current preclinical evidence suggests that pan-target pharmacological blockade of Panx1 channels in PDAC may mediate tumor growth suppression through coordinated perturbation of ATP-dependent autocrine-paracrine signaling networks within the tumor-stromal compartment. The mechanistic roles of Panx1 channel in PDAC has been summarized in Figure 2.

Notwithstanding considerable progress in elucidating Panx1 channel pathophysiology in PDAC, critical knowledge gaps persist in several domains. Firstly, 10Panx is not applicable in clinical treatment. Exploring the feasibility of clinical drugs such as PBN, CBX, or Spironolactone as chemotherapy auxiliary treatment drugs for PDAC is a key direction for achieving clinical translation in PDAC treatment. Secondly, it has been reported that Panx1 inhibitor,10Panx, did not alter the lethality of the chemotherapeutic agent Oxaliplatin in colon cancer HT-29 cells (Di Cesare Mannelli et al., 2015). However, the response to chemotherapeutic agents varies among different cancer cell types. The relationship between Panx1 and resistance to first-line chemotherapy in PDAC (e.g., gemcitabine, mFOLFIRINOX) remains unclear. Investigating whether Panx1 inhibitors can enhance the efficacy of these chemo-therapeutics is therefore warranted. Notably, Panx1 promotes PDAC cell growth via the P2Y2-PI3K/AKT signaling axis (Wang et al., 2020). Supporting this therapeutic approach, *in vivo* targeting of P2RY2 with the selective inhibitor AR-C118925XX significantly inhibited PDAC tumor progression and prolonged survival in orthotopic models (Hu et al., 2019). Importantly, combining AR-C118925XX with gemcitabine demonstrated synergistic benefits (Hu et al., 2019). Thirdly, PI3K/AKT and NF-κB signaling pathways play important roles in PDAC, and Panx1 affects PDAC cell proliferation and immune infiltration through these two pathways. It remains to be seen if Panx1 channel inhibitors could be combined with signaling pathway inhibitors to control PDAC. Fourth, the cell types in the tumor microenvironment of PDAC are varied and complex, and the expression patterns and specific functions of Panx1 in each types of stromal cells remain unclear. Fifth, pain is one of the clinical symptoms in PDAC patients (Porta et al., 2005) and is also a clinical trial endpoint. Opioids are the main drug choice for treating pain in PDAC patients, but they severely burden intestinal function (Coveler et al., 2021). *In vivo* injection of 10Panx alleviates pain sense in tongue cancer (Koyama et al., 2021), and reverses Oxaliplatin-induced neuropathic pain (Di Cesare Mannelli et al., 2015). Whether Panx channel inhibitors also have analgesic functions and thus improve patient wellbeing is a very worth question to explore.

Although Panx2 and Panx3 are also involved in various disease processes, there are no inhibitors or drugs targeting the Panx2 or Panx3 channels at present. Additionally, compared to Panxa2 and Panx3, their localization and function in the cytoplasm have been confirmed years ago, while traces of Panx1 were only recently discovered on the mitochondrial membrane in 2023 (Rusiecka et al., 2023). Whether Panx1 widely localizes to the mitochondria in various cell types and participate in other biological functions warrants further investigation. Nevertheless, the clinical translation of Panx inhibitors requires systematic resolution of outstanding questions regarding target selectivity, pharmacokinetic optimization, and potential compensatory signaling mechanisms.

## Glossary

10Panx: Synthetic peptide inhibitor targeting Panx1 W74 to Y83
4TM: Four-Transmembrane
5-FU: 5-Fluorouracil
AAMRGs: Arachidonic Acid Metabolism Related Genes
AHR: Airway Hyperreactivity
AKI: Acute Kidney Injury
AKT: Protein Kinase B (PKB)
APAP: Acetaminophen
AQP5: Aquaporin5
ATP: Adenosine Triphosphate
BAX: BCL2-Associated X Protein
BCL2: B-cell Lymphoma 2
CAD: C-terminal Autoinhibitory Domain
CaMKII: Ca^2+^/calmodulin-dependent kinase II
CBX: Carbenoxolone
CCDS: Consensus Coding Sequence
CCL2: C-C motif chemokine ligand 2
ccRCC: clear cell renal cell carcinoma
CD39: Ectonucleoside Triphosphate Diphosphohydrolase 1(ENTPD1)
CD73: Ecto-5′-Nucleotidase(NT5E)
CFA: Complete Freund’s Adjuvant
Cryo-EM: Cryo-Electron Microscopy
cSCC: cutaneous Squamous Cell Carcinoma
CTD: C-terminal Domain
Cx: Connexin
DAMPs: Damage Associated Molecular Patterns
DDP: Cisplatin
DMM: Destabilization of Medial Meniscus
ECL1/2: Extracellular Loop 1/2
EMT: Epithelial-Mesenchymal Transition
ER: Endoplasmic Reticulum
ERK1/2: Extracellular Regulated Protein Kinases1/2
ES: Epithelioid Neoplasm
FDA: Food and Drug Administration
FGFR2: Fibroblast growth factor receptor 2
GLI1: Glioma-associated homologue-1
GPCR: G-protein-coupled receptors
HCC: Hepatocellular Carcinoma
HDM: House Dust Mite
I/R injury: Ischemia-Reperfusion injury
IC50: Half Maximal Inhibitory Concentration
IL-1α: Interleukin 1 alpha
IL-1β: Interleukin 1 beta
IL-6: Interleukin 6
IL-8: Interleukin 8
iNOS: Inducible Nitric Oxide Synthase
IP3: Inositol Triphosphate
KO: Knockout
LUAD: Lung Adenocarcinoma
LGG: Low-Grade Glioma
LXRβ: Liver X Receptor beta
MAPK: Mitogen-Activated Protein Kinase
MDS: Myelodysplastic Syndrome
MDSCs: Myeloid-Derived Suppressor Cells
MET: Hepatocyte growth factor receptor
mFOLFIRINOX: Oxaliplatin, Irinotecan, Fluorouracil, Leucovorin
MMP9: Matrix Metalloproteinase 9
mTOR: Mechanistic Target of Rapamycin
NETs: Neutrophil Extracellular Traps
NF-κB: Nuclear Factor kappa-B
NLRP3: NOD-like receptor thermal protein domain associated protein 3
NO: Nitric Oxide
NOD1: Nucleotide-binding Oligomerization Domain-containing protein 1
NRF2: Nuclear Factor Erythroid 2-Related Factor 2
NT5E: Ecto-5′-Nucleotidase (CD73)
NTD: N-terminal Domain
OATs: Organic Anion Transporters
OCTs: Organic Cation Transporters
oncRNA T3p: Orphan non-coding RNA T3p
OXPHOS: Oxidative phosphorylation
P2RX1/P2X1: Purinergic Receptor P2X 1
P2RX4/P2X4: Purinergic Receptor P2X 4
P2RX7/P2X7: Purinergic Receptor P2X 7
P2RY2/P2Y2: Purinergic Receptor P2Y 2
P2Y: Purinergic Receptor P2Y
PBMC: Peripheral Blood Mononuclear Cells
PBN: Probenecid
PDAC: Pancreatic Ductal Adenocarcinoma
PDGFR: Platelet-Derived Growth Factor Receptor
PI3K: Phosphatidylinositol 3-Kinase
PKA: Protein Kinase A
PSGL-1: P-selectin Glycoprotein Ligand-1
PTGS2: Prostaglandin-endoperoxide synthase 2
RIPK1: Receptor-Interacting Protein Kinase 1
ROS: Reactive Oxygen Species
SFK: Src-Family Kinase
SIK: Salt-Inducible Kinase
STATs: Signal Transducers and Activators of Transcription
Teff: T-effector cells
TMD: Transmembrane Domain
TNF-α: Tumor Necrosis Factor alpha
Treg: Regulatory T cells
UQCRC1: Ubiquinol-Cytochrome C Reductase Core Protein 1
URAT1: Urate Transporter 1 (SLC22A12)
UTR: Untranslated Region
Wnt: Wingless-related integration site
